# Aging with board games: fostering well-being in the older population

**DOI:** 10.3389/fpsyg.2024.1501111

**Published:** 2024-11-18

**Authors:** Veronica Guardabassi, Evelyn Manoni, Marta Di Massimo, Elisa Cirilli, Alessandro Maranesi, Paola Nicolini

**Affiliations:** Department of Humanities, University of Macerata, Macerata, Italy

**Keywords:** older people, active aging, positive aging, board games, well-being

## Abstract

**Introduction:**

The increase in the average age of the population has resulted in a greater focus on interventions designed to facilitate successful Ageing. Notwithstanding its potential, the strategy of the board game remains relatively underexplored. This study aims to ascertain its role in fostering older people’s well-being. Specifically, it was hypothesized that the level of well-being associated with the gaming experience is greater than overall well-being, particularly when the level of difficulty is low.

**Methods:**

From an initial number of 164 participants, a total of 132 older people made up the final sample (*M_age_* = 74.05; *SD* = 5.62). They were divided into groups of four or five individuals and engaged in a gaming session of varying levels of difficulty: low (*N* = 44), medium(*N* = 49) and high (*N* = 36). Prior to each game session, participants completed a questionnaire regarding their general well-being. After the game session, they filled out a similar questionnaire regarding their well-being while gaming.

**Results:**

The results showed that the level of well-being experienced while playing was significantly higher than that observed in daily life, *F*_(1,131)_ = 14.604, *p* = 0.000, $\eta^{2}$ = 0.100, particularly with board games with a low or medium level of difficulty, [*F*_(2,126)_ =  = 10.982, *p* = .001, $\eta^{2}$ = 0.148].

**Discussion:**

Board games with an appropriate level of difficulty can be useful tools for promoting wellbeing in the older population. Future studies and possible interventions for people in the third and fourth ages will be discussed.

## 1 Introduction

Aging is a natural, complex, and heterogeneous phenomenon that involves many aspects of human beings (WHO, 2020) and affects an increasing number of people. WHO estimates that by 2050, one in five people in the world will be over 60 years old, and the number of people over 80 will double (WHO, 2022). Life expectancy is increasing worldwide, making it necessary to manage and promote health among the older population.

### 1.1 Aging

Globally, aging is one of the most relevant current challenges from both health (e.g., cognitive decline, medical diseases, Dogra et al., 2022) and socioeconomic perspectives (Belachew et al., 2024). Health complications in older people make it imperative to find adequate and sustainable responses to ensure the well-being and quality of life of older people (Marquez et al., 2020). Aging is associated not only with physical decline but also with social and emotional challenges (Gates et al., 2020). For example, as older people experience reduced physical mobility, they may be at increasing risk of having lower autonomy levels and perceived low control over their lives (Nyende et al., 2023; Ylönen et al., 2024). In addition, they may have lost friends and relatives, be isolated, and have a reduction or lack of positive social relationships, leading to negative feelings and loneliness. These situations can negatively impact perceptions of well-being and quality of life (Abu Elheja et al., 2021). Thus, older people can be at risk of a decline in psychological well-being (Buecker et al., 2023), and interventions to support them and their health are strongly recommended (Luke et al., 2024).

### 1.2 Active aging

In line with these considerations, the active aging perspective recognizes the aging process not only as a physical and cognitive decline but also as an opportunity to optimize individuals’ health and improve quality of life (WHO, 2020) through promoting enjoyment, satisfaction, social fulfillment, and well-being (Goodwin et al., 2023). Indeed, according to the WHO (1948), health is not only the absence of disease but the result of a positive interaction between physical, mental, and social dimensions (Engel, 1977). In other words, health concerns the positive perception of one’s general state of physical, psychological, and relational well-being. Interventions to promote successful aging aim to foster psychophysical well-being by promoting healthy lifestyles, such as good nutrition, physical activity, cognitive stimulation, and social participation (Belachew et al., 2024). Several studies found that living in one’s own home for as long as possible (Ylönen et al., 2024), having autonomy in making life choices, and being able to use urban and extra-urban green spaces contribute to a sense of security, opportunities for social interaction, and well-being in older people (Fowler Davis et al., 2024; Hodgson et al., 2023; Sixsmith et al., 2023). Other research has found that expressive and creative activities such as art, dance, and yoga have positive effects on both cognitive and socio-affective dimensions (Chiang et al., 2024; Crealey et al., 2023; McQuade and O’Sullivan, 2023; Wang et al., 2021; Yang et al., 2021; Azman et al., 2017). The literature highlights that active aging interventions have multiple beneficial outcomes, including improved physical function, cognitive function, mental health, social health, and sleep (Dogra et al., 2022). Active aging interventions are associated with life satisfaction (Marsillas et al., 2017), positive quality of life (Cunningham et al., 2020), good social relationships, and well-being (Bruine de Bruin et al., 2020; Cresswell-Smith et al., 2019). Therefore, research into new strategies to foster well-being for older people is strongly encouraged and recommended, and board games can be used for this purpose.

### 1.3 Game-based activities and well-being

Play has always been an integral part of human culture, so much so that it can be considered one of the most important human experiences, extending well beyond childhood. Engaging in play is vital for physical, mental, and emotional well-being, serving as a powerful stimulus from cognitive, social, emotional, and ethical perspectives (Bruner et al., 1976; Piaget, 1945; Vygotskij, 1934; Winnicott, 1971). In addition, playing is considered a powerful tool to increase individuals’ intrinsic motivation (Caillois, 1958) and achieve a mental state of flow (Csikszentmihalyi, 1975), especially when games are designed with an appropriate level of difficulty (Hattie et al., 2020; Ryan and Deci, 2020; Ryan et al., 2006; Csikszentmihalyi, 1990).

For example, Gerling et al. (2012) suggested that games for older people should be easy to understand to facilitate interaction without adding to cognitive load. In line with this, Skjæret-Maroni et al. (2016) found a slight decrease in performance when participants transitioned from the first to the second difficulty level of an exergame, indicating a potential decline in motivation and an increase in mind wandering (Thomson et al., 2015), which contrasts with the flow state. Furthermore, Chen and Janicki (2020) found that older participants felt proud and satisfied after engaging in a challenging game, emphasizing that personal experiences with board games significantly influence outcomes: mastering a game is positively associated with achieving a state of flow (Hodent, 2017).

For these reasons, adequate game-based activities may be a useful strategy to promote active aging (Aguirre-Cardona and Mendoza-Espinel, 2022).

Most interventions implemented through games are based on digital games (Afridi et al., 2021; Alhasan et al., 2017; Ayed et al., 2019; Campo-Prieto et al., 2021). Literature has shown that digital games have a positive impact on cognitive functioning (Anguera et al., 2021; Bonnechère et al., 2021; Torres, 2011; Hou and Li, 2022) and contribute to reducing feelings of social isolation and symptoms of depression (Cicek et al., 2020; de Morais et al., 2020; Antunes et al., 2017; Khosravi et al., 2016), as well as increasing individuals’ self-efficacy (Czaja et al., 2018) and well-being (Kaufman et al., 2018; Lee et al., 2021; Mclaughlin et al., 2018; Seah et al., 2018).

However, the majority of older people spend their time with non-digital forms of gaming (Mortenson et al., 2017). Studies have shown that non-digital forms of gaming lead to benefits in terms of socialization, quality of life, depression symptoms, and feelings of loneliness (Hallgren et al., 2020; Mortenson et al., 2017; Yu et al., 2023). Within this group of games are the board games. Although they are used more as an extended strategy to counteract cognitive decline and train cognitive functions (Ching-Teng, 2019; Estrada-Plana et al., 2021; Chen and Tsai, 2022) or to reduce negative psychopathological symptoms (e.g., depression, Lee et al., 2020), some studies have investigated the role of board games in promoting physical and psychological health.

Indeed, board games are considered useful tools to improve people’s health (e.g., Nakao, 2019; Gauthier et al., 2019), and positive results have been obtained in older people. Diniz et al. (2022) obtained positive results using a board game to prevent falls in older people, which are very common and dangerous in this population, while Tsai et al. (2024) designed a board game that was effective in increasing knowledge, attitudes and preventive behaviors regarding osteoporosis. Board games are also seen as a way of socializing; older people consider playing an opportunity to spend time and create new social relations (Cousins and Witcher, 2007) or to strengthen social relations (Outley and McKenzie, 2007). Consistent results have also been found by Chen and Tsai (2022); they conducted research aimed at investigating the effectiveness of board games in improving interpersonal communication, interpersonal relationships, and self-efficacy, as well as reducing loneliness. The board game involved four life themes (thank you, sorry, love, and goodbye) and took place in a center for older people for 4 weeks. The results showed significant improvements in interpersonal communication, self-efficacy, and perceived loneliness at the end of the intervention, suggesting that board games should be used in projects aimed at promoting the well-being of older people.

In contrast, Estrada-Plana et al. (2021) found a different result in their study; they conducted a pilot study that included 35 participants who played modern board and card games (experimental group) or performed paper-pencil cognitive tasks (control group) to investigate the effects of board games on executive functions, depressive symptoms and quality of life in healthy older people aged 65 years and over. The intervention sessions took place twice a week for 5 weeks. They showed that board and card games can effectively stimulate cognitive functions but not satisfaction with the health or quality of family relationships. However, according to the authors, the type of game played and different sample compositions may explain the results. More recently, Bodner et al. (2024) investigated the effect of a make-believe game on the well-being and loneliness of older people; they used this board game for 3 months (once a week) in small groups of 4 or 5 people and found that, unlike participants in the control group, older people who played the Kioku board game showed increased well-being and reduced feelings of loneliness.

To conclude, board games may contribute significantly to promoting health, stimulating cognitive functions, and strengthening social relationships among older people. They appear to serve as a valuable coping strategy that can help older individuals manage the challenges associated with aging, such as physical illnesses, cognitive decline, and social isolation, ultimately fostering their overall well-being.

### 1.4 The present study

The older population is constantly increasing, resulting in demographic, epidemiological, and anthropological changes (WHO, 2022). This demographic shift underscores the importance of adopting positive aging experiences. Several programs have been implemented to achieve this goal (Belachew et al., 2024), but few studies have investigated the role of play activities despite their positive effects from a cognitive, social, and emotional point of view (Chen and Tsai, 2022; Mortenson et al., 2017). This study fills this gap in the literature by exploring the role of board games in promoting well-being in older people, a psychological dimension related to positive aging (Bruine de Bruin et al., 2020; Cresswell-Smith et al., 2019).

Evidence from the literature shows that board games promote well-being in children (Dell’Angela et al., 2020; Gashaj et al., 2021; Zaharia et al., 2022) and young adults (Gonzalo-Iglesia et al., 2018; Kloep et al., 2023), and have a positive impact on physical health, cognitive function and socialisation in older people (e.g., Bodner et al., 2024; Chen and Tsai, 2022; Diniz et al., 2022; Lee et al., 2020). Therefore, it was hypothesized that participation in board games would increase well-being in older people, i.e., gaming well-being is significantly higher than general well-being (H1). Furthermore, previous studies have found that a positive game experience is associated with an appropriate level of difficulty (Csikszentmihalyi, 1990; Hattie et al., 2020; Ryan and Deci, 2020; Ryan et al., 2006), especially for games with a low level of difficulty and low cognitive effort (Gerling et al., 2012; Skjæret-Maroni et al., 2016) or with a challenging game (Chen and Janicki, 2020). Thus, it was expected that older people, novice players with low board game skills (Hodent, 2017), would experience high levels of well-being when playing a board game with an easy or medium level of difficulty (H2).

## 2 Methods

### 2.1 Participants and procedure

Data were collected as part of a larger investigation into the psychological and cognitive dimensions of play across the lifespan. The study was approved by the Ethical Commitment of the University of Macerata, and participants consented to take part in the study.

Based on *a priori* sample computation (repeated measure ANOVA: 3 ×2; power test: 0.8; effect size: 0.25; significant level: 0.005; type of effect: interaction effect), a sample of 157 people was planned. Thus, a total of 164 older people and members of Community Centres for the Third Age were invited to participate. Specifically, they were aged 65 years or over, according to the definition of older people by ISS (2014), and they were regular and active participants in the Community Centre’s activities, with no experience with board games (people with a diagnosis of dementia were not included in this study). Two team researchers went to the Community Centre (*N* = 12) to implement the study. They first introduced the research to inform older adults, obtained their consent to participate, and explained the data handling process. Then, the researchers invited the participants to form small groups of 4–5 people and sit around small tables to start the research session (1.5 h).

At the beginning, the participants filled out a questionnaire about their personal information and general well-being. Then, each group was given a board game to play. Three board games with different levels of complexity were used. Participants in the low-complexity condition played a game of chance, i.e., a game with simple rules and dependent on luck (*N* = 51). The medium complexity level had skill or learning games with rules that required participants to memorize new words, pay attention to symbols, or guess a name using deductions (*N* = 57). Participants who played the high-complexity game (*N* = 52) had a strategy game with rules such as working together to solve logical-mathematical puzzles, choosing the better way to play according to the situation, or acquiring clues to continue the game. At the end, participants answered a questionnaire about their well-being during the game and expressed their satisfaction with the experience. All older adults participated in the entire study session, but some participants did not answer all questions of the well-being questionnaire. Thus, the final sample consisted of 132 older people (*M_age_* = 74.05 years old, *SD* = 5.62; *Min_age_* = 65; *Max_age_* = 89) who played the board game of low (*N* = 44), medium (*N* = 49), and high (*N* = 36) difficulty.

### 2.2 Measures

#### 2.2.1 Information about participants

To obtain general information about the participants, data were collected on gender, year of birth, nationality, retirement status (not retired; <1 year; <5 years; for 5 years; more than 5 years), marital status (single, married, divorced, widow/widower), number of sons/daughters and grandchildren. The level of education was also examined (primary school, middle school, high school, bachelor’s, master’s, postgraduate specialization). This variable was categorized as “educational level” with primary school equating to 5 years of education. Secondary school corresponded to 8 years of education and so on.

#### 2.2.2 General well-being

The WHO-5 questionnaire (WHO, 1998) is a unidimensional measure of psychological well-being that refers to participants’ experiences over the last 2 weeks. It consists of five items: (1) I have felt cheerful and in good spirits; (2) I have felt calm and relaxed; (3) I have felt active and vigorous; (4) I woke up feeling fresh and rested; (5) My daily life has been filled with things that interest me. Participants indicate their level of agreement using a 6-point Likert scale, ranging from 0 (never) to 5 (always). The final score corresponds to the mean of each response, and a higher score corresponds to a better assessment of one’s well-being. Cronbach’s alpha was 0.857.

#### 2.2.3 Gaming well-being

Game-related well-being results from an adaptation of the WHO-5 questionnaire (WHO, 1998). It proposes the same five questions about the participants’ feelings during the game: (1) I have felt cheerful and in good spirits; (2) I have felt calm and relaxed; (3) I have felt active and vigorous; (4) I have felt fresh and relaxed; (5) The game has been filled with things that interest me. Participants answered by using the 6-point Likert scale of WHO-5 (WHO, 1998), and the same scoring method was adopted. The reliability of the questionnaire was equal to 0.872.

#### 2.2.4 Satisfaction questionnaire

The Satisfaction Questionnaire was an *ad hoc* instrument designed to explore participants’ level of satisfaction with their gaming experience. Using a Likert scale ranging from 1 (strongly disagree) to 5 (strongly agree), participants expressed their level of agreement with seven statements about feeling competent, having fun, enjoying spending time with friends, age appropriateness of the activity, interest, gratitude, and wanting to repeat the experience. The score on the satisfaction questionnaire is the mean score on the scale, which has a reliability of 0.592.

### 2.3 Data analyses

Statistical analyses were performed using SPSS 25. Descriptive and correlational analyses were performed between the main variables, and an ANOVA was conducted to know about group differences. Repeated-measures ANOVA was used to identify differences between participants’ general well-being and gaming well-being. ANOVA analysis was also used to investigate differences in satisfaction according to the game’s complexity level.

## 3 Results

### 3.1 Descriptive and correlational data

Among the 132 participants (88 women, 44 men), 129 were Italian, while 2.3% had not provided the information. Regarding retirement status, 9.8% had been retired for 1 year, 7.6% for <5 years, 2.3% for 5 years, while 58.3% had been retired for more than 5 years (9.1% were not retired and 12.2% were missing). Responses regarding marital status indicated that 6.1% of participants were single, 53.8% were married, 7.6% were divorced, and 21.2% were widows/widowers. Table 1 summarizes the means and bivariate correlations between the main variables.

**Table 1:** Descriptive data and correlational analyses.

	*M* (*SD*)	Bivariate correlations					
		2	3	4	5	6	7
1. Age	74.05 (5.62)	−0.289**	0.198*	0.163	−0.143	−0.137	−0.146
2. Educational level	10.42 (4.37)	–	−0.027	−0.001	0.036	0.071	0.040
3. Son/Daughter	1.50 (0.98)	–	–	0.177	0.058	−0.040	−0.056
4. Grandchildren	1.32(1.43)	–	–	-	−0.010	−0.173	−0.379**
5. General well-being	2.82 (1.15)	–	–	–	–	0.164	0.125
6. Gaming well-being	3.37 (1.41)	–	–	–	–	–	0.370**
7. Satisfaction	3.90 (0.47)	–	–	–	–	–	–

*Significant at the level of 0.05 (2-tails) **Significant at the level of 0.01 (2-tails).

Concerning group composition (see Table 2), ANOVA analyses showed no differences among groups, except for participants’ age [*F*_(2, 126)_ = 3.987, *p* = 0.021]. Specifically, participants who played with a low level of difficulty were older (*M* = 75.81; *SD* = 5.78) than participants who were in the highly difficult board game (*M* = 72.66; *SD* = 5.33; *p* = 0.031).

**Table 2:** Group composition: means, standard deviations, and mean differences.

	Level of difficulty				
	Low	Medium	High		
	*M (SD)*	*M (SD)*	*M (SD)*	*F*	*p*
Age	75.81 (5.78)	73.32 (5.06)	72.66 (5.33)	3.978	0.021
Educational level	10.59 (4.79)	9.50 (4.03)	11.75 (4.07)	2.622	0.077
Son/Daughter	1.78 (0.96)	1.26 (1.00)	1.56 (0.97143)	2.763	0.068
Grandchildren	1.41 (1.49)	1.24 (1.46)	1.36 (1.40156)	0.140	0.869

*Significant at the level of 0.05 (2-tails) **Significant at the level of 0.01 (2-tails).

### 3.2 Well-being and gaming well-being

Repeated-measures ANOVA showed that general well-being (*M* = 2.82; *SD* = 1.15) was significantly lower than gaming well-being (*M* = 3.37; *SD* = 1.41) [*F*_(1, 131)_ = 14.604, *p* = 0.000, *η*^2^ = 0.100].

An effect of board-game level of complexity was also found [*F*_(1, 126)_ = 10.982, *p* = 0.000, *η*^2^ = 0.148]. Indeed, post-hoc analyses suggest that participants who played board games of easy, *F*_(1, 126)_ = 13.967, *p* = 0.000, *η*^2^ = 0.098, or medium, *F*_(1, 126)_ = 22.208, *p* = 0.000, *η*^2^ = 0.150, level of difficulty presented a gaming well-being significantly higher than general well-being. This difference was not significant in participants with highly difficult board games [*F*_(1, 126)_ = 3.128, *p* = 0.079, *η*^2^ = 0.024]. Table 3 describes these results.

**Table 3:** Gaming well-being and level of difficulty.

	General well-being		Gaming well-being		*MD*	*F*	*p*	*η* ^2^
	*M*	*SD*	*M*	*SD*				
Playing with board games	2.82	1.15	3.37	1.41	+0.488	14.604	0.000	0.100
Playing and game-level								
Low level	2.81	1.27	3.69	1.18	+0.873	13.967	0.000	0.098
Medium level	2.75	1.16	3.81	1.85	+1.053	22.208	0.000	0.150
High level	2.90	1.06	2.43	1.48	−0.461	3.128	0.079	0.024

MD, mean difference.

In addition, post-hoc analyses revealed that the general well-being of participants in easy (*M* = 2.81; *SD* = 1.27), medium (*M* = 2.75; *SD* = 1.16), and high (*M* = 2.90; *SD* = 1.06) levels of difficulty was not significantly different [*F*_(2, 126)_ = 0.148, *p* = 0.862, *η*^2^ = 0.002]. Instead, participants’ gaming well-being changes according to the level of game difficulty [*F*_(2, 126)_ = 13.459, *p* = 0.000, *η*^2^ = 0.176]. Results showed that participants who played a game of high level of difficulty had a level of gaming well-being (*M* = 2.43; *SD* = 1.48) lower than that of older people who played with medium (*M* = 3.81; *SD* = 1.85; *p* = 0.000) or easy levels of game difficulty (*M* = 3.69; *SD* = 1.18; *p* = 0.000). No differences in gaming well-being were found between participants who played with easy and medium levels of difficulty (*p* = 0.653).

Repeated analysis of ANOVA, controlling for age and educational level, revealed that well-being level did not significantly change from general to gaming experience: *F_well-being_* (1,119) = 0.012, *p* = 0.915, *η*^2^ = 0.000; *F_ageXwell-being_* (1,119) = 0.09, *p* = 0.925, *p*^2^ = 0.000; *F_educationalLevelXwell-being_* (1,119) = 1.22, *p* = 0.270, *η*^2^ = 0.010. However, the effect of board game level of difficulty was significant, *F*_(2,119)_ = 11.740, *p* = 0.000, *η*^2^ = 0.165, and post-hoc analyses showed significant effects (see Table 4).

**Table 4:** Well-being and level of difficulty: *post-hoc* analyses.

	General well-being		Gaming well-being		*MD*	*F*	*p*	*η^2^*
	*M*	*SD*	*M*	*SD*				
Low level	2.81	1.27	3.69	1.18	+0.873	13.967	0.001	0.096
Medium level	2.76	1.17	3.80	1.26	+1.001	22.208	0.000	0.152
High level	2.87	1.12	2.31	1.50	−0.616	3.128	0.031	0.038

MD, mean difference.

Gaming well-being was higher than general well-being when the board game had an easy, *F*_(1,119)_ = 12.647, *p* = 0.001, *η*^2^ = 0.096, or medium level of difficulty, *F*_(1,119)_ = 21.375, *p* = 0.000, *η*^2^ = 0.152, whereas it was significantly lower with a high level of difficulty [*F*_(1, 119)_ = 4.669, *p* = 0.033, *η*^2^ = 0.038]. Furthermore, gaming well-being significantly changed with board game difficulty level, *F*_(2, 119)_ = 17.294, *p* = 0.000, *η*^2^ = 0.225: gaming well-being reported by participants who played with high difficult games was significantly lower compared to the other two groups (*p_low level_* = 0.000; *p_medium level_* = 0.000).

### 3.3 Satisfaction and board game type

ANOVA analyses showed that participants who played high-difficulty games reported the highest satisfaction with the experience [*F*_(2, 86)_ = 3.178, *p* = 0.047]. Their satisfaction (*M* = 4.08; *SD* = 0.47) was higher than participants in medium (*M* = 3.84; *SD* = 0.38) and low (*M* = 3.78; *SD* = 0.43) game level conditions. In addition, post-hoc analyses showed no variance between medium and low levels of difficulty (*p* = 0.600) but significant differences between participants who played with the high levels of difficulty compared to the medium (*p* = 0.021) or low levels (*p* = 0.044).

## 4 Discussion

This study investigated the role of board games as a viable tool to promote the well-being of older people and, consequently, to promote positive aging. Confirming the hypotheses of this study, the results showed that well-being during a single game was higher than general well-being, especially when board games had a low or medium level of difficulty.

The literature on play activities has provided evidence of the impact of board games on psychological well-being. Indeed, several studies have investigated the role of gaming experiences on children’s emotional development (Zaharia et al., 2022; Dell’Angela et al., 2020), university students’ well-being (Guardabassi et al., 2024) or adults’ flow state (Khan and Pearce, 2015; Kloep et al., 2023). However, there is very little research on older people. Furthermore, the majority of studies on older people and play activities have mainly focused on cognitive performance (Ching-Teng, 2019; Estrada-Plana et al., 2021; Chen and Tsai, 2022), and the few investigations on the effects of board games on psychological health have shown contrasting results. For example, Estrada-Plana et al. (2021) found no significant differences in depressive symptoms and quality of life after weeks of board game sessions, whereas results from Chen and Tsai (2022), Bodner et al. (2024), or Lee et al. (2020) suggested that board games improved interpersonal communication, self-efficacy, and well-being and reduced depression symptoms in older people. This study integrates these contradictions by showing that the positive effects of board games depend on the level of complexity of the board game.

Moreover, in line with previous studies that emphasized the importance of game complexity level to motivate participants (Hattie et al., 2020; Ryan and Deci, 2020; Ryan et al., 2006), to have an optimal game experience (Csikszentmihalyi, 1990), board games are only effective when the level of complexity is not high. Just as simple digital games are more effective in older participants (Gerling et al., 2012; Skjæret-Maroni et al., 2016), older people who played the low-difficulty board game showed a higher gaming well-being than general well-being. Indeed, people who played a board game for the first time could mostly benefit from an easy and comfortable game (Hodent, 2017). However, older people also enjoyed playing a board game with a medium level of difficulty. Similar to the digital puzzle in Chen and Janicki’s (2020) study, it probably represents a good mix of difficulty and the opportunity to gradually grasp the game’s goals. The most difficult board game, with complex problem-solving activities, was perceived to be beyond the participants’ possibility [i.e. beyond the zone of proximal development (Vygotskij, 1934)], and less enjoyable to play.

The results of this study have also enriched the literature on positive aging (e.g., Belachew et al., 2024), especially the area dealing with active sedentary activities (e.g., Hallgren et al., 2020; Kikuchi et al., 2014). In addition to physical or recreational activities (Dogra et al., 2022), board games with an appropriate level of difficulty can be included in positive aging programs for the well-being of older people. The results also suggest that participants were satisfied with their gaming experience, particularly older people who played the most difficult board game and reported lower levels of gaming well-being. This difference could be explained by cognitive dissonance theory (Festinger, 1962), but it should also be understood by considering gaming well-being and participant satisfaction as two different dimensions.

Although this study filled a gap in the literature and offered a new perspective on positive aging, its limitations should be considered. Firstly, no group tested a condition without any game-based activities or experienced the game activity in a different context. However, three different game conditions make it possible to understand the phenomenon and formulate considerations regarding board game types. Second, the mean age of participants in the game groups was different. Nevertheless, in the most challenging conditions, there were younger people who should feel less threatened by the level of difficulty, according to ageism stereotypes (Kang and Kim, 2022). Despite this, they were the most negatively affected. Thirdly, the final sample was less numerous due to missing data, and smaller groups reduced the test power. In addition, groups in the final sample did not have the same number of participants. Specifically, the high-difficulty condition has the largest number of missing responses, as 10 older people did not complete the final questionnaire correctly. However, despite the fewer participants in this condition, this behavior may represent another index of boredom or low motivation induced by high-difficulty games. Fourthly, the gaming well-being and satisfaction questionnaires were not standardized or measures used in previous studies. Nevertheless, the reliability of the gaming well-being scale is satisfactory, and the satisfaction questionnaire was used for descriptive purposes. Fifth, gaming well-being corresponds more to hedonic well-being, i.e., physical and emotional pleasure, than to eudemonic well-being, i.e., satisfaction and consistency with one’s values (Huta and Ryan, 2010). Although hedonic well-being is useful in reducing negative emotions such as depression or stress (Henderson et al., 2013), which is critical in older people (Hu et al., 2022), there is no evidence of its effects over the long term. Sixth, there was no manipulation check after the game experiences to verify the perception of the level of difficulty. However, the research team organized 14 telephonic interviews and two debriefing groups to better understand the study’s results. Participants considered the game with a high level of difficulty too complicated for older people; the game with a medium level was evaluated as difficult but interesting, whereas the easy game was considered a little childish.

Future studies can overcome these limitations and develop new research designs. For example, longitudinal studies can provide an opportunity to explore the long-term effects of game-based experiences on well-being and to extend the findings of Bodner et al. (2024) by investigating the role of different types of board games. Other unexplored dimensions such as social relationships, life satisfaction, or health-related quality of life may also be new unknown outcomes. Gaming activities are also related to psychological satisfaction, flow state, and cognitive stimulation (Errity et al., 2016). These outcomes should also be investigated in the older population. Otherwise, action research can be useful in developing board games in collaboration with older people: products should be able to respond to older people’s needs and be specific to their age. For example, Fernandes et al. (2023) created a board game called “The Ark of Rights” with older people, and the game successfully empowered older people regarding their rights. Similar research may be useful in identifying new strategies to promote the well-being of older people. The motivations and interests of older people should not be underestimated. Indeed, the game’s content and people’s interests may moderate the relationships between board games and older people’s well-being, and future studies should also focus on these variables.

The results are particularly important because board games can be a low-cost intervention to promote the well-being of older people. They can be easily adopted by social community centers, and playing board games can become a routine practice. Similarly, board games can also be used in family or other social contexts, as they provide a common platform for younger and older people, and intergenerational activities have a positive impact on the health of individuals (Cès et al., 2024; Canedo-Garcia et al., 2017) and on age-related attitudes (WHO, 2023). However, given the importance of choosing the right board game with an appropriate level of difficulty, play educators represent a very important professional figure: they can be beneficiaries of specific training programs and leaders of board game activities with older people as players.

In conclusion, board games can effectively promote positive aging, provided that an appropriate difficulty level is selected. Offering board games with varying and progressively challenging difficulty levels could enhance engagement, and involving experts in game selection and facilitation would further optimize these activities for older adults.

## Data Availability

The raw data supporting the conclusions of this article will be made available by the authors without undue reservation.
